# CEA-DETR: A Multi-Scale Feature Fusion-Based Method for Wind Turbine Blade Surface Defect Detection

**DOI:** 10.3390/s26072115

**Published:** 2026-03-28

**Authors:** Xudong Luo, Ruimin Wang, Jianhui Zhang, Junjie Zeng, Xiaohang Cai

**Affiliations:** 1 School of Cyberspace Security, Zhengzhou University, Zhengzhou 450002, China; luoxd0219@gs.zzu.edu.cn (X.L.); zengjj_lab@163.com (J.Z.); caixxxh@163.com (X.C.); 2 School of Computer Science and Artificial Intelligence, Zhengzhou University, Zhengzhou 450001, China; iermwang@zzu.edu.cn; 3 Songshan Laboratory, Zhengzhou 450046, China

**Keywords:** wind turbine blade, defect detection, multi-scale features, RTDETR, attention mechanism

## Abstract

Wind turbine blade surface defect detection remains challenging due to large variations in defect scales, blurred edge textures, and severe interference from complex backgrounds, which often lead to insufficient detection accuracy and high false and missed detection rates. To address these issues, this paper proposes an improved RTDETR-based detection framework, termed CEA-DETR, for wind turbine blade surface defect inspection. First, a Cross-Scale Multi-Edge feature Extraction (CSME) backbone is designed by integrating multi-scale pooling and edge-enhancement units with a dual-domain feature selection mechanism, enabling effective extraction of fine-grained texture and edge features across different scales. Second, an Efficient Multi-Scale Feature Fusion Network (EMSFFN) is constructed to facilitate deep cross-level feature interaction through adaptive weighted fusion and multi-scale convolutional structures, thereby enhancing the representation of multi-scale defects. Furthermore, an adaptive sparse self-attention mechanism is introduced to reconstruct the AIFI module, strengthening global dependency modeling and guiding the network to focus on critical defect regions under complex background conditions. Experimental results demonstrate that CEA-DETR achieves mAP50 and mAP50:95 of 89.4% and 68.9%, respectively, representing improvements of 3.1% and 6.5% over the RT-DETR-r18 baseline. Meanwhile, the proposed model reduces computational cost (GFLOPs) by 20.1% and parameter count by 8.1%. These advantages make CEA-DETR more suitable for deployment on resource-constrained unmanned aerial vehicles (UAVs), enabling efficient and real-time autonomous inspection of wind turbine blades.

## 1. Introduction

Against the background of continuously growing global energy demand and the accelerated transition of energy structures, wind energy has become an essential pillar of modern power systems due to its clean and renewable nature [1,2].

As the core component responsible for converting wind energy into electrical power, the operational condition of wind turbine blades directly affects power generation efficiency and service life. However, during long-term operation, wind turbine blades are subjected to complex cyclic loads and harsh environmental conditions, which makes them highly susceptible to surface defects such as cracks, corrosion, and skin debonding [3]. If these defects are not detected and repaired in a timely manner, they may lead to aerodynamic performance degradation or even catastrophic structural failures, resulting in severe economic losses and safety hazards [4,5]. Therefore, the development of efficient and accurate defect detection techniques for wind turbine blades is of great engineering significance for ensuring the safe and reliable operation of wind farms throughout their life cycle.

Traditional wind turbine blade defect detection methods mainly include visual inspection, ultrasonic testing, vibration monitoring, thermographic inspection, and acoustic emission monitoring [6]. For instance, Choung et al. [7] proposed an adaptive phased-array ultrasonic inspection platform for detecting internal blade defects; Tcherniak et al. [8] developed a vibration-based structural health monitoring system; Hwang et al. [9] employed laser scanning thermography to detect internal delamination in wind turbine blades; and Xu et al. [10] utilized acoustic emission techniques to identify blade damage modes. Although these methods can effectively enhance blade safety to a certain extent, they usually rely on specialized equipment and manual interpretation, suffering from low inspection efficiency, high operational costs, and strong subjectivity. As a result, they are difficult to scale for large-scale and periodic inspections in wind farms.

In recent years, deep learning-based object detection techniques have provided new opportunities for wind turbine blade defect detection. Two-stage detectors such as Faster R-CNN [11] and Mask R-CNN [12] achieve high detection accuracy but suffer from relatively slow inference speeds. In contrast, single-stage detectors, including SSD [13] and the YOLO family [14,15,16,17,18], exhibit superior real-time performance; however, they typically rely on non-maximum suppression (NMS) for post-processing, which introduces additional computational overhead in dense-object or complex-background scenarios. Moreover, wind turbine blade defects are characterized by large scale variations, blurred edges, and severe background interference. Traditional convolutional neural networks (CNNs), due to their limited local receptive fields, have inherent limitations in capturing global contextual information. Here, contextual information refers to the holistic scene understanding that helps associate defects with their surrounding environment. For example, local crack or corrosion regions need to be interpreted in relation to the overall blade structure and distinguished from complex background interferences such as vegetation, mountains, or shadows. When defects are extremely small or partially occluded by complex textures, the lack of global perception can degrade detection performance. In contrast, Transformer-based architectures [19], by leveraging self-attention mechanisms, possess a natural advantage in modeling long-range dependencies and global context. The DETR model proposed by Carion et al. [20] introduced an end-to-end object detection paradigm that eliminates anchor design and NMS post-processing. Nevertheless, DETR suffers from slow convergence and high computational costs. To address these issues, Baidu proposed RT-DETR [21], which significantly improves inference speed through efficient encoding structures and feature interaction strategies while preserving the advantages of end-to-end detection, achieving a favorable balance between accuracy and efficiency.

Despite its promising performance in general object detection tasks, the original RT-DETR architecture still exhibits limitations when applied to wind turbine blade defect detection. First, its multiscale feature fusion capability is insufficient to effectively handle defects with large scale variations. Second, existing Transformer-based architectures mainly rely on global self-attention mechanisms, which tend to prioritize long-range contextual information while sacrificing high-frequency local details [22]. This characteristic limits the model’s ability to represent high-frequency edge features, making it difficult to accurately localize fine-grained defects with weak boundaries and blurred edges. Third, under complex background conditions, the attention mechanism is prone to being disturbed by redundant information. To address these challenges, this paper proposes a wind turbine blade surface defect detection model based on RT-DETR, termed CEA-DETR. The main contributions of this work are summarized as follows:

1. A Cross-Scale Multi-Edge feature extraction backbone (CSME) is designed, which captures spatial details and global semantic information under different receptive fields through multiscale pooling. By integrating an edge information enhancement unit and a dual-domain selection mechanism, the proposed backbone effectively strengthens the representation of weak edges and high-frequency texture features, thereby improving the model’s multiscale feature extraction capability.
2. An Efficient Multiscale Feature Fusion Network (EMSFFN) is constructed. By adopting an adaptive weighted fusion strategy, deep interactions among multi-level features are achieved. Combined with efficient upsampling and multiscale convolution modules, the spatial resolution of the feature pyramid is reconstructed and the receptive field is expanded, facilitating complete transmission and spatiotemporal alignment of high-frequency texture details and global semantic information. As a result, the model’s perception ability for defects of different scales and its detection robustness are significantly enhanced.
3. An adaptive sparse self-attention (ASSA) mechanism is introduced to redesign the AIFI structure, resulting in the proposed ASSA-AIFI module. This module employs a dynamic sparsification strategy to adaptively adjust the distribution of attention weights, effectively suppressing redundant responses from irrelevant regions while preserving global contextual information. Consequently, the model focuses more on critical defect regions, leading to a substantial improvement in feature discrimination capability under complex background conditions.

## 2. Related Work

### 2.1. CNN-Based Object Detection Methods

CNN-based object detection methods have long dominated industrial visual inspection applications and can generally be categorized into two groups: two-stage detectors and one-stage detectors.

Two-stage detection algorithms are centered on the region proposal network (RPN). They first generate candidate regions and then perform classification and bounding box regression on these proposals. R-CNN [23] was the first to introduce deep learning into object detection by generating region proposals through selective search and extracting features using CNNs. Fast R-CNN [24] significantly improved detection efficiency by sharing convolutional feature maps. Faster R-CNN [11] further introduced the RPN to enable end-to-end training, establishing a classical paradigm for two-stage detection frameworks. Based on this architecture, Mask R-CNN [12] added an instance segmentation branch, achieving joint learning of object detection and segmentation. Although two-stage detectors generally achieve high detection accuracy, their inference speed is relatively slow due to the requirement of multiple forward passes.

One-stage detection algorithms directly perform object classification and localization on feature maps without the need for region proposal generation. YOLO [25] was the first to formulate object detection as a regression problem, enabling real-time detection. Subsequently, YOLOv5–v12 [14,15,16,17,18] have continuously improved detection performance by incorporating feature pyramid structures, attention mechanisms, and efficient convolutional modules. SSD [13] adopts multi-scale feature maps for detection, effectively improving small object detection performance. One-stage detectors exhibit significant advantages in real-time applications; however, they usually rely on NMS for post-processing, which may affect detection stability in dense-object or heavily occluded scenarios.

In the field of wind turbine blade defect detection, CNN-based object detection methods have been widely investigated. Pratt et al. [26] employed multiple CNN backbones to construct a Mask R-CNN-based framework and improved defect detection performance through a multi-model voting strategy. Qiu et al. [27] enhanced multiscale object recognition capability by constructing a feature-enhanced image pyramid. Liu et al. [28] introduced lightweight modules and model distillation strategies into YOLOv10s, achieving improved detection accuracy while reducing computational complexity. Ma et al. [29] proposed the MES-YOLOv8n model, which reduced the number of parameters by approximately 30% while achieving a 1.7% improvement in detection accuracy. Fu et al. [30] improved detection accuracy by 4.2% through the introduction of GSConv and the SimAM attention module. Zhang et al. [31] proposed the GCB-YOLO model, which optimizes the backbone network by integrating coordinate attention and GhostNet modules. Tong et al. [32] further improved detection efficiency with the WTBD-YOLOv8 approach.

Although the aforementioned methods have achieved notable progress in wind turbine blade defect detection, most existing models still rely on NMS for post-processing. In complex background conditions or defect-dense scenarios, the associated computational overhead and sensitivity to threshold selection may adversely affect detection stability and real-time performance. Moreover, conventional CNN-based models are inherently limited by local receptive fields, resulting in insufficient modeling capability for long-range dependencies between defects. Consequently, there remains significant room for improvement in detection performance under scenarios involving multi-scale defect coexistence and severe background interference.

### 2.2. Transformer-Based Object Detection Methods

The Transformer architecture [19] was originally proposed for natural language processing, where its self-attention mechanism enables effective modeling of global dependencies among sequence elements. In recent years, Transformers have been introduced into the field of computer vision and have demonstrated unique advantages in object detection tasks. DETR [20] was the first to apply Transformers to object detection by combining CNN-based feature extraction with an encoder–decoder Transformer architecture, achieving end-to-end object detection without relying on anchor design or NMS-based post-processing. However, DETR suffers from slow convergence and suboptimal performance in small object detection.

To address these limitations, numerous improved variants have been proposed. Deformable DETR [33] introduces a deformable attention mechanism that significantly reduces computational complexity and accelerates convergence through sparse sampling strategies. Conditional DETR [34] improves detection efficiency by optimizing query design via conditional cross-attention. DINO [35] further enhances detection performance on multiple benchmark datasets by incorporating contrastive denoising training and hybrid query selection strategies.

For real-time detection scenarios, RT-DETR [21] was proposed to overcome the low inference speed of DETR-based models. RT-DETR employs an efficient hybrid encoder to rapidly fuse multi-scale features and adopts an uncertainty-minimization-based query selection strategy to improve decoding efficiency. As a result, RT-DETR achieves real-time inference while preserving the advantages of end-to-end detection, striking a favorable balance between detection accuracy and inference speed. Compared with traditional YOLO-based detectors that rely on local receptive fields, RT-DETR demonstrates stronger global modeling capability and eliminates the need for NMS post-processing. Therefore, RT-DETR is selected as the baseline framework in this study.

Nevertheless, RT-DETR is primarily designed for general object detection in natural scenes and still exhibits certain limitations when applied to wind turbine blade surface defect detection. Blade defects are characterized by large scale variations, irregular morphologies, low edge contrast, and complex background textures, which impose higher requirements on multi-scale feature modeling, edge information representation, and global feature interaction. In addition, many blade defects, such as cracks and slight spalling, exhibit weak edge contrast and ambiguous boundaries, making them difficult to distinguish from surrounding textures. Although Transformer-based architectures provide stronger global contextual modeling capabilities, they still heavily depend on the quality of extracted features. When edge features are weakened or degraded during deep feature extraction, the attention mechanism may fail to accurately focus on defect boundaries, leading to missed detections of fine-grained defects. Therefore, the original RT-DETR architecture leaves room for improvement in multi-scale feature fusion and local detail enhancement.

Based on the above analysis, this study proposes the CEA-DETR model by introducing targeted improvements to the RT-DETR framework from three perspectives: feature extraction, feature fusion, and attention modeling, in order to better accommodate the visual characteristics of wind turbine blade defects.

## 3. Method Overview

**Figure 1:**
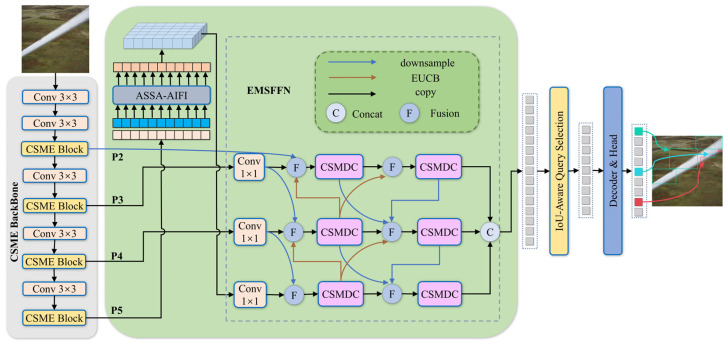
Structure diagram of CEA-DETR: CSME block denotes the core module of the CSME backbone network; ASSA-AIFI represents the improved feature interaction module; CSMDC stands for the cross-stage partial multi-scale depthwise convolution module; Downsample indicates downsampling; EUCB is the efficient upsampling convolution module; Copy represents direct feature copying and transmission; Concat denotes the concatenation operation; Fusion refers to the fast normalized fusion method.

To address the challenges of wind turbine blade surface defect detection, including large variations in defect scales, blurred edge textures, and complex background interference, this paper proposes an improved RT-DETR-based detection framework termed CEA-DETR. The overall network architecture is illustrated in Figure 1. The proposed model mainly consists of three key components: the CSME backbone, the ASSA-AIFI module, and the EMSFFN feature fusion network.

The overall workflow of the proposed CEA-DETR model is as follows. First, the input wind turbine blade images are fed into the feature extraction backbone CSME, which is composed of a series of convolutional layers and alternately stacked CSME blocks. Through hierarchical feature extraction, the backbone generates multi-scale feature maps denoted as $P_{2}$, $P_{3}$, $P_{4}$, and $P_{5}$.

Next, the highest-level feature map $P_{5}$ is processed by the ASSA-AIFI module, where an adaptive sparse self-attention mechanism is employed to enable efficient intra-scale feature interaction and global context modeling. Subsequently, the refined $P_{5}$ feature map, together with the lower-level feature maps $P_{2}$, $P_{3}$, and $P_{4}$, is fed into the EMSFFN to perform adaptive multi-scale feature fusion.

Finally, the fused multi-scale features are delivered to the decoder, where an IoU-aware query selection mechanism is applied to generate the final wind turbine blade defect detection results.

### 3.1. CSME

In wind turbine blade defect detection tasks, the large variation in defect scales and complex background interference pose significant challenges to feature extraction. Although recent efficient visual backbones, such as EfficientViT [36] and MambaOut [37], have achieved promising performance in general vision tasks by introducing lightweight attention mechanisms or state space modeling strategies, they primarily focus on global semantic modeling and computational efficiency optimization. As a result, their capability in capturing fine-grained structural information remains limited when applied to industrial defect detection scenarios characterized by weak texture features and complex background interference.

Specifically, defects such as cracks, peeling, and corrosion on wind turbine blades typically exhibit blurred edges, irregular shapes, and significant scale variations. During feature extraction, conventional backbone networks tend to suffer from two major limitations. On the one hand, repeated convolutional downsampling operations gradually lead to the loss of fine-grained edge information, thereby weakening the model’s ability to perceive small-scale defects. On the other hand, fixed convolutional structures lack sufficient adaptability for multi-scale feature modeling, making it difficult to simultaneously preserve local detailed information and high-level semantic representations. This limitation further affects the robustness of detection models under complex background conditions.

To address these issues, the backbone network is redesigned by deeply integrating a Multi-scale Edge Information Extraction Module (MEIEM) with the Cross Stage Partial (CSP) structure [38]. Based on this design, a novel CSME Block is constructed and employed as the fundamental building unit of the proposed CSME feature extraction backbone. The key novelty of CSME lies in its joint enhancement of edge-aware representation and dual-domain feature selection. Instead of relying solely on global attention or lightweight modeling strategies, CSME explicitly strengthens high-frequency edge information while simultaneously integrating spatial and frequency-domain features. This design enables the network to better capture subtle structural variations and weak defect boundaries that are often overlooked by conventional backbones.

**Figure 2:**
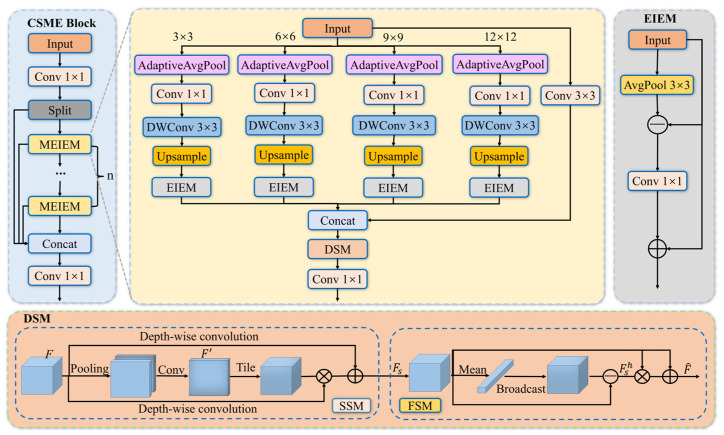
Structure diagram of CSME Block: MEIEM denotes the Multi-scale Edge Information Extraction Module; AdaptiveAvgPool represents the adaptive average pooling operation; DWConv stands for the depthwise separable convolution operation; Upsample indicates the upsampling module; EIEM refers to the Edge Information Enhancement Module; Concat denotes the feature concatenation operation; DSM represents the Dual-Domain Feature Selection Mechanism; AvgPool indicates the average pooling operation.

As illustrated in Figure 2, the CSME Block splits the input feature map into two parallel branches. One branch is processed through multiple cascaded MEIEM modules for enhanced feature extraction, while the other branch directly preserves and propagates the original features. The outputs of the two branches are then concatenated and fused via convolutional operations. This design effectively alleviates the progressive loss of fine-grained details caused by deep convolutional stacking, while preserving spatial continuity and low-level semantic information.

Specifically, within the MEIEM module, in order to simultaneously capture local details and global contextual information, the feature extraction process is decomposed into one local convolution branch and four parallel multi-scale branches. Given the input feature $X\in R^{C\times H\times W}$, the local branch employs a 3 × 3 standard convolution to preserve spatial detail information:

(1) $$X_{local}=Conv_{3\times 3}\left(X\right)$$

For the multi-scale branches, adaptive average pooling with different output sizes is applied to capture contextual information under multiple receptive fields. The pooling sizes are set to 3 × 3, 6 × 6, 9 × 9, and 12 × 12, respectively. For the i-th branch, the feature is first compressed by a 1 × 1 convolution to reduce channel dimensionality, followed by a 3 × 3 depthwise convolution to perform spatial feature encoding with low computational cost. The encoded low-resolution features are then upsampled to the original spatial resolution H ×W using bilinear interpolation. The process can be formulated as:

(2) $$F_{i}^{up}=UpSample\left(DWC_{3\times 3}\left(Conv_{1\times 1}\left(AdaptiveAvgPool_{b_{i}}\left(X\right)\right)\right),\left(H,W\right)\right)$$

where $b_{i}\in \{3,6,9,12\}$ denotes the pooling size, and $UpSample\left(\cdot \right)$ represents bilinear interpolation.

To further enhance defect edges and suppress background interference, each upsampled feature $F_{i}^{up}$ is subsequently fed into the Edge Information Enhancement Module (EIEM). The EIEM module is specifically designed to strengthen defect edge features by extracting high-frequency information while suppressing background texture interference, which is particularly important for detecting small-scale and edge-sensitive defects such as cracks and lightning strikes. Concretely, EIEM first applies local average pooling to the input feature X to obtain a low-frequency background representation. The high-frequency edge response is then computed by subtracting the pooled feature from the original input. To further enhance salient edge information and reduce noise, the EIEM performs a convolution operation with a Sigmoid activation function on the edge information and fuses it with the input features to generate enhanced output features, thereby improving the response intensity of features in edge regions. The calculation process is as follows:

(3) $$E_{i}=F_{i}^{up}+\sigma \left(Conv\left(F_{i}^{up}-AvgPool_{3\times 3}\left(F_{i}^{up}\right)\right)\right)$$

where σ denotes the Sigmoid activation function, and $E_{i}\in R^{\frac{C}{4}\times H\times W}$ represents the enhanced edge features at the i-th scale.

Subsequently, the output of the local branch $X_{local}$ is concatenated with the enhanced edge features from four scales along the channel dimension to obtain the fused features $F_{local}\in R^{2C\times H\times W}$:

(4) $$F=Concat\left(X_{local},E_{1},E_{2},E_{3},E_{4}\right)$$

Since multi-scale features obtained by simple concatenation may contain excessive redundant high-frequency noise, this study further introduces the Dual-Domain Selection Mechanism (DSM) [39] to screen out the key edge regions of defects while filtering out ultra-high-frequency noise, outputting refined features with continuous and complete edges. Specifically, the DSM module adaptively screens the fused multi-scale edge features F in both frequency and spatial domains, suppressing redundancy and highlighting useful features. The DSM consists of two components: the Spatial Selection Module (SSM) and the Frequency Selection Module (FSM). The overall calculation process is as follows:

(5) $$\hat{F}=FSM(SSM(F))$$

where $F\;and\;\hat{F}$ denote the input and output feature maps of the DSM, respectively.

The SSM aims to identify and focus on severely degraded regions in the spatial domain, thereby providing location-aware guidance for subsequent frequency-domain processing. Specifically, average pooling and max pooling are first applied along the channel dimension to compress the input feature, followed by a convolution operation to generate a spatial attention map $F^{\prime }$:

(6) $$F^{\prime }=Conv(AvgPool(F),MaxPool(F))$$

Subsequently, to model degradation variations across different channels, the SSM employs multi-scale depthwise separable convolutions to perform channel-wise transformations. Combined with the spatial attention map, the spatially selected feature map $F_{s}$ is obtained as:

(7) $$F_{s}={\mathrm{DConv}}_{5,7}(F)⊗T(F^{\prime };C)+{\mathrm{DConv}}_{3}(F)$$

where ⊗ denotes element-wise multiplication, ${DConv}_{5,7}(\cdot )$ represents cascaded depthwise convolution layers with kernel sizes of 5 × 5 and 7 × 7, respectively, and T(⋅;C) is a replication operation that expands $F^{\prime }$ along the channel dimension to match the size of F.

The FSM further refines feature representations by enhancing critical high-frequency information while suppressing redundant low-frequency components. Specifically, global average pooling is first applied to extract the low-frequency component of the input feature, and the complementary high-frequency signal is computed as:

(8) $$F_{s}^{h}=F_{s}-Mean(F_{s})$$

where Mean(⋅) denotes global average pooling along the channel dimension, and $F_{s}^{h}$ represents high-frequency features containing key edge and texture information.

Finally, element-wise multiplication is employed to amplify high-frequency responses, and a residual connection is introduced to preserve effective information from the spatial selection stage, resulting in the refined output feature:

(9) $$\hat{F}=F_{s}^{h}⊗F_{s}+F_{s}$$

Overall, by further optimizing and filtering the edge-enhanced fused features generated by the EIEM module, the DSM significantly improves feature robustness and noise suppression capability, thereby effectively enhancing the overall performance of wind turbine blade defect detection.

### 3.2. EMSFFN

The Cross-scale Feature Fusion Module (CCFM) in the original RT-DETR model effectively integrates multi-level feature maps extracted from the backbone network, enabling the capture of multi-scale information and achieving promising performance in conventional object detection scenarios. However, when applied to wind turbine blade defect detection tasks characterized by large scale variations and strong background interference, CCFM still exhibits certain limitations. First, although CCFM employs the RepC3 module to facilitate multi-scale feature interaction, it typically relies on single-scale convolution operations during feature alignment and fusion, making it difficult to simultaneously capture fine-grained local details and long-range contextual information within the same feature level. Second, traditional upsampling operations commonly adopt simple interpolation or standard convolution, lacking fine-grained modeling of high-resolution textures, which can easily lead to blurred edges of small-scale defects and the loss of high-frequency texture information. In addition, CCFM usually fuses features from different levels through straightforward summation or concatenation, which fails to effectively distinguish the relative importance of different features, potentially resulting in insufficient utilization of critical information or the introduction of redundant background noise. To address these issues, this paper proposes the EMSFFN module.

Compared with conventional multi-scale feature fusion structures, such as FPN [40], PANet [41], and BiFPN [42], the proposed EMSFFN introduces targeted improvements in both feature interaction and fusion mechanisms. Traditional feature fusion methods typically rely on fixed top-down or bidirectional pathways for information propagation and adopt simple operations such as element-wise addition or concatenation for feature aggregation. However, such strategies are prone to introducing redundant background information in complex industrial scenarios and are insufficient for modeling the discrepancies among features at different scales.

To address these limitations, EMSFFN incorporates a more effective feature reconstruction and fusion strategy during cross-scale interaction. First, an Efficient Upsampling Convolution Block (EUCB) [43] is designed, which integrates depthwise separable convolution and channel reorganization during the upsampling process. This design enhances the spatial representation capability of high-level semantic features while promoting sufficient inter-channel interaction, thereby compensating for the limitations of traditional interpolation-based upsampling in recovering fine details. Second, a Multi-scale Depthwise Convolution Module (MDCM) is introduced in the feature fusion stage. By employing parallel depthwise convolutions with different receptive fields, MDCM enables simultaneous modeling of local detailed features and global contextual information within the same feature level, thus improving the representation capability for multi-scale defect targets. Furthermore, the multi-scale depthwise convolution structure is embedded into a CSP framework to construct the Cross-Stage Multi-Scale Depthwise Convolution (CSMDC) module. This design enhances feature reuse while reducing computational redundancy, enabling more efficient and stable information flow across different feature levels. Compared with conventional fusion structures that rely on repeated full-channel convolution operations, the proposed approach effectively reduces computational complexity while improving feature fusion efficiency through the combination of depthwise separable convolution and cross-stage partial connections.

**Figure 3:**
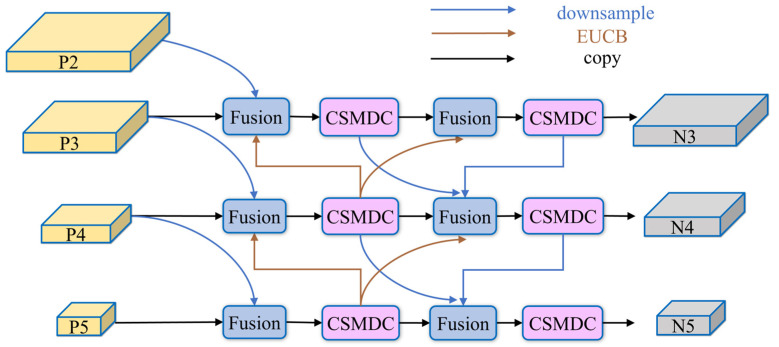
Structure diagram of EMSFFN: Fusion refers to the fast normalized fusion method; CSMDC denotes the cross-stage partial multi-scale depthwise convolution module; Downsample represents downsampling; EUCB indicates the efficient upsampling convolution module; Copy means direct feature copying and transmission.

As illustrated in Figure 3, EMSFFN takes four feature maps at different scales, denoted as $P_{2}$, $P_{3}$, $P_{4}$, and $P_{5}$, generated by the CSME backbone network as inputs. These feature maps contain both shallow spatial details and deep semantic representations. To enhance cross-scale feature interaction, EMSFFN constructs a bidirectional multi-path feature pyramid topology, breaking the limitation of unidirectional information flow in conventional FPN structures and enabling efficient circulation and interaction between shallow and deep features.

To resolve the feature weight allocation problem during fusion, EMSFFN adopts the adaptive weighted feature fusion mechanism from BiFPN. This mechanism receives lateral features from the same level, upsampled deep semantic features, and downsampled shallow detail features, and assigns learnable weights to different input features. By dynamically adjusting the contribution of each feature branch according to image content, the BiFPN-based fusion strategy effectively suppresses background noise while significantly enhancing defect responses on wind turbine blades, thereby strengthening cross-scale semantic consistency.

**Figure 4:**
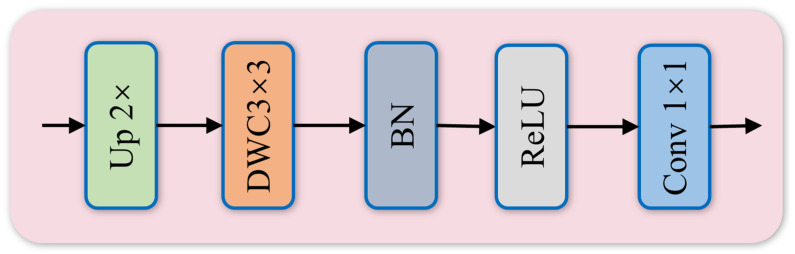
Architecture of EUCB: Up denotes the upsampling operation; DWC represents the depthwise separable convolution module; BN indicates the batch normalization operation; ReLU stands for the ReLU activation function.

In the top-down pathway of the feature pyramid, to more precisely preserve critical feature information and alleviate aliasing effects introduced by upsampling, EUCB is incorporated, as shown in Figure 4. Different from direct interpolation or standard deconvolution, EUCB performs feature reconstruction in a spatially and channel-wise decoupled manner. The module first upsamples the input feature map $X\in R^{C\times H\times W}$ by a scale factor of 2 via interpolation to preliminarily restore spatial resolution. Subsequently, a 3 × 3 depthwise separable convolution is applied to the upsampled feature map for spatial filtering and edge detail reconstruction. Finally, after batch normalization and the ReLU activation function, a 1 × 1 convolution is adopted to complete the linear combination of channel dimensions to match the channel requirements of subsequent operations. The computational formulation of this process can be expressed as:

(10) $$X_{up}\;=DWC_{3\times 3}\;(Upsample(X))$$

(11) $$X_{out}\;=Conv_{1\times 1}\;(ReLU(BN(X_{up}\;)))$$

where Upsample denotes the upsampling operation, DWC represents depthwise separable convolution, BN stands for batch normalization, and ReLU refers to the ReLU activation function.

Through this design, EUCB effectively restores high-resolution edge textures with minimal computational overhead, thereby alleviating the blurring problem of small-scale defect features during scale transformation.

**Figure 5:**
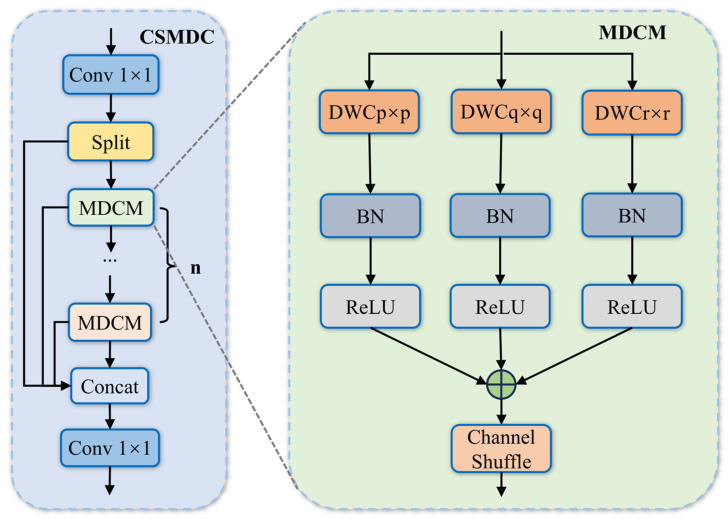
Architecture of CSMDC.: MDCM denotes the Multi-scale Depth Convolution Module; Concat denotes the feature concatenation operation; DWC represents the depthwise separable convolution module; BN indicates the batch normalization operation; ReLU stands for the ReLU activation function; Channel shuffle refers to the channel shuffle operation.

Furthermore, to enhance the representation capability of multi-scale features and optimize inference efficiency, the CSMDC module is designed, as illustrated in Figure 5. The input feature map is first divided into two parts: one part is directly propagated without modification, while the other part is processed by multiple MDCM modules. The two parts are then concatenated to produce the output feature.

Within each MDCM, a parallel multi-branch structure is adopted, where the input feature is fed into three parallel branches equipped with depthwise convolution kernels of different sizes. This design constructs multi-granularity receptive fields and enables adaptive capture of defects at different scales. The features from each branch are activated by batch normalization and ReLU functions, and then fused through element-wise summation to aggregate multi-receptive-field information into a unified feature space.

Since depthwise convolution operates independently within each channel and may weaken inter-channel interactions, a channel shuffle operation is introduced after fusion to reorganize channel distributions. This promotes effective interaction among semantic information from different branches, yielding enhanced features with both multi-scale perception and cross-channel correlation. The computational process is formulated as:

(12) $$Y=f_{shuffle}(\sum_{k\in \{p,q,r\}}\mathrm{ReLU}(BN(DWC_{k\times k}(X))))$$

where X denotes the input feature map, ${DWC}_{k\times k}$ represents depthwise convolution with kernel size k, and $f_{\mathrm{shuffle}}$ denotes the channel shuffle operation.

In summary, through the collaborative optimization of upsampling schemes, multi-scale feature modeling, and feature fusion structures, EMSFFN reduces invalid feature transmission and redundant computation, while enhancing the effective interaction capability between multi-scale features. Consequently, it significantly improves the detection performance of the model for wind turbine blade defects in complex background environments.

### 3.3. ASSA-AIFI

In the baseline RT-DETR model, the Attention-based Intra-scale Feature Interaction (AIFI) module is applied to high-level features to capture rich global contextual information. However, the standard dense multi-head self-attention mechanism computes pairwise interactions among all spatial positions. In the context of wind turbine blade defect detection, input images often contain large areas of intact blade surfaces and complex natural backgrounds such as sky and vegetation. Such dense interactions not only introduce substantial computational overhead but also inevitably incorporate irrelevant background noise into defect representations, thereby degrading detection accuracy. To address these challenges and further enhance global context modeling in high-level semantic feature encoding, this paper reconstructs the AIFI module in the RT-DETR encoder by introducing an Adaptive Sparse Self-Attention (ASSA) mechanism [44], resulting in the proposed ASSA-AIFI module.

**Figure 6:**
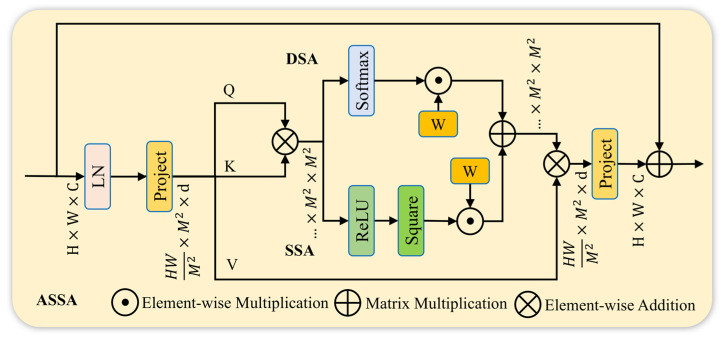
Architecture of ASSA: LN denotes the layer normalization operation; Project represents the linear projection layer; softmax stands for the standard softmax normalization function; ReLU indicates the ReLU activation function; square refers to the square operation; W represents the learnable dynamic weight.

As illustrated in Figure 6, the ASSA module takes a feature map of dimension C × H × W as input. The input feature is first normalized along the channel dimension using layer normalization to stabilize feature distributions. Subsequently, the feature map is partitioned into non-overlapping M × M windows and flattened, followed by linear projection to generate the query (Q), key (K), and value (V) representations.

Next, attention weights are computed through parallel Dense Self-Attention (DSA) and Sparse Self-Attention (SSA) branches based on the dot-product of Q and K. Specifically, to preserve the global receptive field and prevent information loss, the DSA branch adopts standard Softmax normalization, retaining all Q–K pairwise interactions to ensure the completeness of global contextual information. In contrast, to eliminate interference from irrelevant background regions in wind turbine blade images, the SSA branch adopts the squared ReLU activation function instead of the traditional Softmax normalization. Leveraging its nonlinear property, it automatically suppresses low-correlation redundant interactions with negative Q-K pair matching scores, retaining only high-response weights in salient regions and reducing redundant computations.

To address potential information loss caused by excessive sparsity, the outputs of the two branches are dynamically fused via an adaptive fusion mechanism. The fusion process is formulated as:

(13) $$A=(w_{1}\cdot {\mathrm{ReLU}}^{2}(\frac{QK^{T}}{\sqrt{d}}+B)+w_{2}\cdot \mathrm{Softmax}(\frac{QK^{T}}{\sqrt{d}}+B))V$$

where A denotes the fused attention output, $w_{1}$ and $w_{2}$ are learnable weights, B represents a learnable relative positional bias, and d denotes the feature dimension. The fused attention map A is then multiplied with the value matrix V, followed by a linear projection to restore the channel dimension. Finally, a residual connection is applied by adding the projected output to the original input feature, producing the final output of the ASSA-AIFI module.

By transforming dense fully connected attention into a task-adaptive sparse attention mechanism, the ASSA-AIFI module enables more efficient feature interaction during the encoding stage. This design effectively suppresses interference from irrelevant regions while reducing redundancy in attention allocation under limited computational overhead. Consequently, computational resources are more focused on modeling features from critical defect regions, significantly enhancing the model’s capability to represent fine-grained defects under complex background conditions. As a result, the proposed ASSA-AIFI module achieves an excellent balance between detection accuracy and feature interaction efficiency.

Moreover, the dual-branch adaptive fusion mechanism dynamically regulates the attention focus range, further mitigating feature redundancy and noise propagation. This not only improves detection accuracy for fine-grained defects in complex industrial scenes but also enhances overall computational resource utilization, making the proposed approach well-suited for real-time wind turbine blade defect detection tasks.

## 4. Experimental Results and Analysis

### 4.1. Dataset

**Figure 7:**
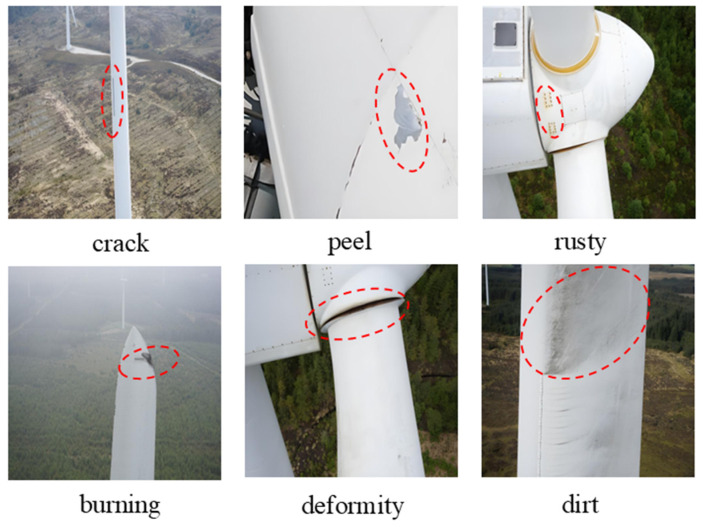
Examples of various defect images in the dataset. Defects highlighted in red dot circle.

Due to the lack of publicly available professional datasets for wind turbine blade surface defect detection, and considering the high diversity of defects observed in real-world operating conditions, this study constructs a dedicated wind turbine blade defect dataset by collecting images captured during UAV-based inspections in wind farms. To comprehensively cover various defect types and improve the generalization capability of the model, offline data augmentation techniques, including image flipping, random cropping, and exposure adjustment, were applied to the collected images. As a result, a dataset containing 4468 images with a resolution of 640 × 640 pixels was obtained. The constructed dataset covers six common types of defects encountered in practical applications: crack, burning, peel, deformity, rusty, and dirt (as illustrated in Figure 7). For data annotation and dataset construction, all defect instances were manually annotated using the LabelImg tool with bounding boxes. The annotation files were saved in the standard YOLO format to ensure compatibility with mainstream deep learning frameworks. Finally, the entire dataset was randomly divided into training, validation, and test sets with a ratio of 7:2:1, which were used for model training, hyperparameter tuning, and final performance evaluation, respectively.

### 4.2. Experimental Setup

All experiments were conducted under a unified hardware and software environment. The operating system was Ubuntu 22.04, equipped with an Intel^®^ Xeon^®^ Platinum 8255C CPU and 43 GB of system memory. An NVIDIA RTX 3090 GPU with 24 GB of video memory was used for model training and inference. The proposed method was implemented using the PyTorch deep learning framework (version 2.1.2), with Python 3.10 and CUDA 11.8. During training, the batch size was set to 16, and the AdamW optimizer was adopted. All input images were resized to 640 × 640 pixels. The initial learning rate was set to 1 × 10^−4^, with a weight decay coefficient of 1 × 10^−4^. The model was trained for 200 epochs to ensure stable convergence.

### 4.3. Evaluation Metrics

To comprehensively evaluate the detection performance and computational efficiency of the proposed method, several widely adopted metrics in object detection were employed, including Precision (P), Recall (R), Average Precision (AP), mean Average Precision (mAP), the number of parameters (Params), and computational complexity measured by GFLOPs.

Precision and Recall are defined as:

(14) $$P=\frac{TP}{TP+FP}$$

(15) $$R=\frac{TP}{TP+FN}$$

where TP, FP, and FN denote the numbers of true positives, false positives, and false negatives, respectively. Precision reflects the reliability of the detected defect samples, while Recall measures the model’s ability to identify all defect instances.

Average Precision (AP) is computed as the area under the Precision–Recall (P–R) curve for each defect category, which reflects the overall detection performance by jointly considering Precision and Recall across different confidence thresholds. The mean Average Precision (mAP) is calculated by averaging AP values over all defect categories:

(16) $$mAP=\frac{1}{N}\sum_{i=1}^{N}AP_{i}$$

where N denotes the total number of defect classes.

In addition to detection accuracy, this study also provides a comprehensive evaluation of model complexity and practical inference efficiency. The number of parameters (Params) is used to measure the model size and memory consumption, while the computational complexity is quantified by the number of floating-point operations (GFLOPs). Furthermore, to explicitly assess the model’s suitability for real-time industrial applications, such as deployment on UAV platforms, frames per second (FPS) is introduced as an evaluation metric. FPS represents the number of image frames that the model can process per second under the same hardware conditions, serving as a direct and critical indicator of real-time deployment capability.

### 4.4. Comparison Experiment of Backbone

To verify the effectiveness of the proposed CSME feature extraction backbone, comparative experiments were conducted by replacing the backbone network of RT-DETR with several representative mainstream backbones, including the original ResNet18 and ResNet50, as well as FasterNet [45], MambaOut [37], Swin Transformer [46], EfficientViT [36], and MobileNetV4 [47]. During the experiments, only the backbone network was changed while all other training settings and model configurations were kept identical.

As shown in Table 1, the proposed CSME backbone achieves superior performance across multiple evaluation metrics. Specifically, CSME attains a Precision of 89.5%, a Recall of 85.9%, and an mAP50 of 88.2%, outperforming all the compared backbone networks. In comparison, ResNet50 and Swin Transformer achieve mAP50 values of 88.0% and 87.9%, respectively. Although these backbones exhibit competitive performance, they are still slightly inferior to CSME.

In terms of model complexity, CSME also demonstrates a favorable balance between accuracy and efficiency. The parameter size of CSME is only 15.8 MB, which is significantly smaller than that of ResNet50 (43.1 MB) and MambaOut (15.9 MB), while achieving higher detection accuracy. This indicates that CSME can effectively enhance feature representation without introducing excessive model complexity.

Some lightweight backbones, such as FasterNet and EfficientViT, exhibit advantages in terms of parameter size; however, this efficiency is achieved at the cost of detection performance. For instance, the model with MambaOut as the backbone yields the lowest mAP50 of 83.4% among all compared methods. Similarly, although EfficientViT-based models maintain relatively low parameter counts, their mAP50 reaches only 86.6%, which remains inferior to that of CSME.

Overall, these results demonstrate that the proposed CSME backbone significantly improves detection accuracy while maintaining a relatively low computational overhead. This confirms the effectiveness and necessity of CSME for wind turbine blade surface defect detection, particularly in scenarios requiring a balanced trade-off between detection performance and computational efficiency.

### 4.5. Comparison Experiment of Feature Fusion Networks

To verify the effectiveness of EMSFFN in addressing the multi-scale characteristics of wind turbine blade defects, comparative experiments were conducted by adopting CCFM, SlimNeck [48], BiFPN [42], GDNeck [49], MAFPN [50], and EMSFFN as the feature fusion networks, while keeping the backbone network and decoder unchanged. All models were evaluated on the same dataset under identical training strategies and evaluation metrics. The experimental results are summarized in Table 2.

The comparison results indicate that EMSFFN achieves superior performance across multiple metrics, with Precision, Recall, and mAP50 reaching 88.4%, 86.3%, and 87.6%, respectively, outperforming all other compared models. Although SlimNeck exhibits the lowest number of parameters, its detection accuracy is relatively limited. BiFPN maintains a moderate parameter scale; however, its detection performance remains insufficient for the target task. GDNeck and MAFPN achieve a relatively good balance between detection accuracy and computational efficiency, yet their overall performance is still inferior to that of EMSFFN.

By jointly considering detection accuracy and computational efficiency, EMSFFN demonstrates the most favorable performance trade-off, thereby validating the effectiveness of the proposed multi-scale feature fusion strategy.

### 4.6. Comparison Experiment of Attention Module

To verify the performance advantages of the proposed ASSA mechanism when integrated into the AIFI module, comparative experiments were conducted against four mainstream attention mechanisms, including HiLo [51], EAA [52], DHSA [53], and Pola [54]. During the experiments, only the attention mechanism within the AIFI module was replaced, while all other network structures were kept unchanged. All models were evaluated on the same dataset under identical training strategies and evaluation metrics, and the experimental results are reported in Table 3.

As shown by the comparative results, ASSA achieves the best overall performance across all key evaluation metrics. Specifically, in terms of detection accuracy, ASSA attains an mAP50 of 87.4%, outperforming HiLo, EAA, DHSA, and Pola by 1.0%, 1.2%, 1.6%, and 0.7%, respectively. These results indicate that, by incorporating sparse sampling and adaptive weight allocation strategies, ASSA is able to more effectively focus on critical defect regions in wind turbine blade images while suppressing redundant background information, thereby improving overall detection accuracy.

In terms of Precision and Recall, ASSA achieves values of 88.2% and 86.2%, respectively, both of which are superior to those of the compared methods. Compared with the second-best Pola mechanism, ASSA improves Precision and Recall by 0.4 and 0.8 percentage points, respectively. This demonstrates that ASSA not only reduces false detections but also enhances the identification of fine-grained and weakly salient defect targets, which is particularly important for safety-critical applications such as wind turbine blade surface defect inspection.

Regarding model complexity, the model incorporating ASSA contains 20.7 MB of parameters, which is only approximately 0.6 MB higher than that of HiLo, the most lightweight method (20.1 MB). Considering the notable improvement in detection accuracy, this additional computational cost is reasonable and acceptable. Overall, the experimental results demonstrate that ASSA effectively enhances the feature modeling capability of the AIFI module without significantly increasing model complexity, thereby validating the effectiveness and rationality of the proposed ASSA-AIFI design for wind turbine blade surface defect detection tasks.

### 4.7. Comparative Experiment

To comprehensively verify the effectiveness of the proposed CEA-DETR model in improving wind turbine blade defect detection performance, comparative experiments were conducted against several representative state-of-the-art object detection models. These include the two-stage detector Faster R-CNN, single-stage detectors YOLOv5m, YOLOv7, YOLOv8m, YOLOv11m, YOLOv12m. In addition, the Transformer-based Deformable DETR, RTDETR-r50 with a deeper backbone network, and the baseline model RTDETR-r18 are also included. All models were trained and evaluated on the same dataset under identical experimental settings. The quantitative results are reported in Table 4.

As shown in Table 4, the proposed CEA-DETR consistently outperforms mainstream object detection models on the wind turbine blade defect detection task. Specifically, CEA-DETR achieves an mAP50 of 89.4% and an mAP50:95 of 68.9%, while maintaining a compact model size of 15.9 MB and a computational complexity of 52.4 GFLOPs. In addition, the model attains a real-time inference speed of 63.2 FPS, indicating its strong capability in balancing detection accuracy and computational efficiency. Compared with the two-stage detector Faster R-CNN, CEA-DETR yields substantially higher average precision across all defect categories, while significantly reducing both model parameters and computational cost.

When compared with single-stage detectors such as YOLOv7, YOLOv8m, YOLOv11m, and YOLOv12m, CEA-DETR achieves notable performance gains while simultaneously reducing model complexity. In particular, the mAP50 of CEA-DETR is improved by 5.1%, 3.5%, 4.2%, and 4.5%, respectively, with fewer parameters and lower GFLOPs. Compared with YOLOv5m, although CEA-DETR introduces a slightly higher computational cost, it improves mAP50 and mAP50:95 by 4.7% and 8.7%, respectively, along with consistent improvements in other evaluation metrics.

Furthermore, to further validate the superiority of the proposed method within transformer-based detection frameworks, we additionally introduce a larger-scale RT-DETR-r50 model and the classical Deformable DETR model for comparison. The experimental results show that although RT-DETR-r50 and Deformable DETR have significantly larger model sizes and computational costs, their detection accuracy is still inferior to that of the proposed CEA-RTDETR. Specifically, the parameter size and computational cost of RT-DETR-r50 reach 42.1 MB and 136.3 GFLOPs, respectively, while Deformable DETR requires up to 173.2 GFLOPs. However, their mAP50 values are 87.1% and 86.5%, both lower than that of CEA-RTDETR. In addition, due to their larger model scales, the inference speeds of these two models are significantly reduced, achieving only 37.6 FPS and 33.2 FPS, respectively. In contrast, CEA-RTDETR achieves higher detection accuracy while maintaining lower computational complexity, further demonstrating the effectiveness of the proposed architectural design.

Compared with the baseline RT-DETR-r18, CEA-DETR reduces the model size by 4.0 MB, while achieving substantial performance improvements across all six defect categories, including crack, burning, peel, deformity, rusty, and dirt. Specifically, the AP50 improvements are 3.6%, 3.1%, 5.3%, 0.3%, 4.8%, and 1.5%, respectively. Notably, more pronounced gains are observed for peel and rusty defects, which exhibit significant scale variation, indicating that CEA-DETR possesses a stronger capability for learning and representing multi-scale defect features. Furthermore, CEA-DETR improves the more stringent mAP50:95 metric by 6.5%, demonstrating robust detection performance across different IoU thresholds. It is worth noting that although this paper introduces additional feature fusion and adaptive attention modules, which results in a slight and reasonable reduction in the model’s inference speed compared with the baseline model (65.5 FPS), the speed of 63.2 FPS still ensures excellent real-time performance.

Overall, the comparative experimental results demonstrate that the proposed CEA-DETR model successfully achieves an effective balance between detection accuracy and computational efficiency, and exhibits superior performance for wind turbine blade surface defect detection.

### 4.8. Detection Results Comparison

**Figure 8:**
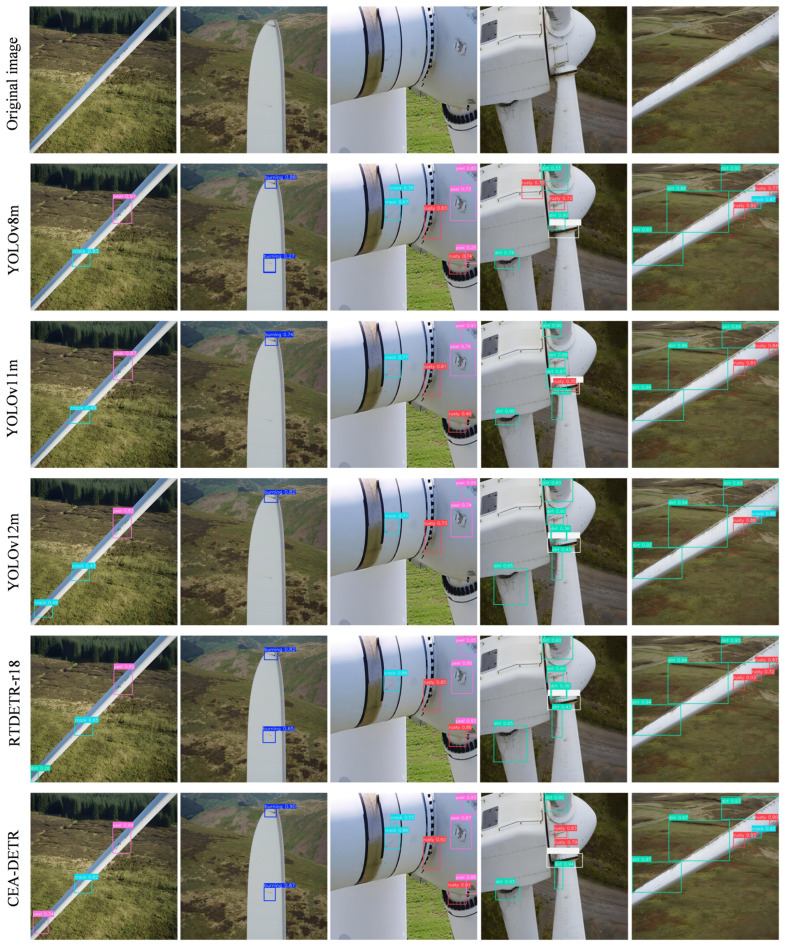
Comparison of Detection Box Effects.

To visually demonstrate the practical detection performance of the proposed CEA-DETR model in wind turbine blade surface defect detection, YOLOv8m, YOLOv11m, YOLOv12m, and the baseline RT-DETR-r18 model, which exhibited relatively strong performance in the previous comparative experiments, were selected as baseline methods for visual comparison. Detection results under various typical scenarios are presented in Figure 8.

From the overall detection results, the YOLO-series models are capable of achieving basic detection performance in scenes with simple backgrounds or large-scale defects. However, under complex natural background conditions, they still suffer from varying degrees of missed detections and false positives. In particular, when the blade surface is affected by complex texture interference such as grassland or mountainous backgrounds, YOLOv8m, YOLOv11m, and YOLOv12m exhibit limited localization accuracy for small-scale and fine-grained defects, such as cracks and slight peeling. In these cases, some predicted bounding boxes show low confidence scores and noticeable localization offsets.

In comparison, the baseline RT-DETR-r18 model demonstrates improved overall detection stability. Nevertheless, in scenarios where small-scale and multi-scale defects coexist, it still shows insufficient sensitivity to weak edge defects, resulting in incomplete detection of fine cracks and mild burning regions. This indicates that relying solely on the original feature extraction and fusion structures is still insufficient for fully modeling fine-grained defect characteristics in complex industrial environments.

The proposed CEA-DETR model exhibits clear advantages in the aforementioned scenarios. First, in small-scale defect detection, CEA-DETR can accurately identify fine cracks and early-stage peeling regions on blade surfaces, with more precise bounding box localization and significantly higher confidence scores compared to the baseline models. Second, in complex scenes with coexisting multi-scale defects, CEA-DETR is able to simultaneously detect large-area rust defects as well as localized cracks and peeling, effectively alleviating interference between features at different scales. Finally, under conditions with strong background interference, CEA-DETR effectively suppresses false detections caused by non-target regions such as grass and shadows, producing predictions that are more concentrated on true defect regions and exhibiting greater overall stability. These results clearly demonstrate the effectiveness of the proposed improvement modules in enhancing detection performance.

In summary, the comparative detection results clearly demonstrate that CEA-DETR outperforms the comparison models in terms of small-scale defect detection accuracy, multi-scale defect perception capability, and robustness under complex background conditions. These results fully validate the effectiveness and practical value of the proposed multi-module collaborative improvement strategy for wind turbine blade surface defect detection.

### 4.9. Heatmap Visualization Analysis

**Figure 9:**
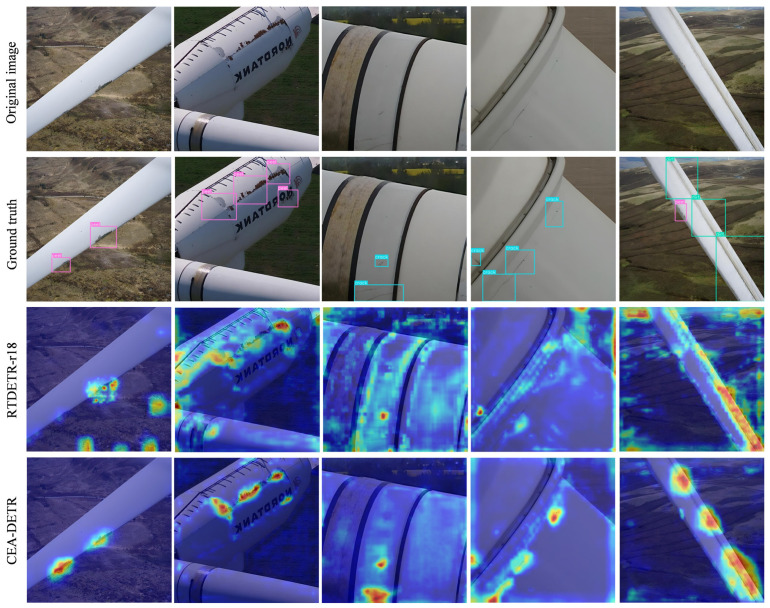
Heatmap Comparison.

To further investigate the feature localization capability and attention distribution of the models, Gradient-weighted Class Activation Mapping (Grad-CAM) was applied to both the baseline RT-DETR-r18 model and the improved CEA-DETR using the same test samples. The resulting heatmaps are shown in Figure 9. As observed, under complex background conditions, the feature responses of the baseline RT-DETR-r18 model are relatively dispersed. Its high-response regions not only cover defect areas but also extend to background regions outside the blade boundaries, while the response intensity at small-scale defects such as cracks and peeling remains relatively weak, making it difficult to form continuous and stable attention regions.

In contrast, CEA-DETR exhibits more concentrated and discriminative feature responses, accurately focusing on the core defect regions while maintaining high sensitivity to defect contours and boundaries. Strong and continuous activation regions are formed along crack edges, peeling areas, and rust defects, while feature responses in non-defect background regions are significantly suppressed, resulting in a clearer and more targeted attention distribution. These observations further confirm that the proposed improvement modules effectively enhance the model’s defect detection capability, reduce missed detections and false positives, and significantly improve its ability to suppress background interference.

### 4.10. Ablation Experiment

#### 4.10.1. Ablation Analysis of the DSM Module

To further validate the effectiveness of the DSM module in the proposed CSME backbone, we conduct a systematic ablation study on its two submodules, namely the Spatial Selection Module (SSM) and the Frequency Selection Module (FSM). The experimental results are presented in Table 5.

When the DSM module is not included in the CSME backbone, the model achieves a precision (P) of 88.7%, recall (R) of 84.3%, and mAP50 of 87.1%, with 14.9 MB parameters and 50.3 GFLOPs. After introducing only the SSM module, the model performance shows a slight improvement, with P, R, and mAP50 increasing to 88.9%, 84.7%, and 87.3%, respectively. This indicates that the spatial selection mechanism enhances the model’s ability to focus on informative spatial regions.

When the FSM module is further incorporated, the model performance improves significantly, achieving P, R, and mAP50 of 89.5%, 85.9%, and 88.2%, respectively. This demonstrates that frequency-domain feature selection can capture complementary information that is difficult to obtain in the spatial domain, thereby further enhancing feature representation capability.

Overall, the combination of SSM and FSM achieves the best performance, indicating that spatial and frequency domain feature selection mechanisms are highly complementary. This combination significantly improves defect detection performance under complex background conditions while introducing only a marginal increase in computational cost. These results verify the effectiveness of the DSM module in enhancing the performance of the proposed CSME backbone.

#### 4.10.2. Ablation Analysis of CEA-DETR

To evaluate the effectiveness of each proposed component, systematic ablation experiments are conducted based on the baseline RT-DETR-r18 model. Specifically, we incrementally introduce the CSME backbone, the EMSFFN feature fusion module, and the ASSA-AIFI attention module to analyze their individual contributions as well as their combined effects. The experimental results are shown in Table 6.

From the perspective of individual module contributions, replacing the original backbone with the proposed CSME significantly improves performance, with precision increasing from 87.3% to 89.5%, recall from 83.4% to 85.9%, and mAP50 from 86.3% to 88.2%. Meanwhile, the number of parameters is reduced from 19.9 MB to 15.1 MB, and GFLOPs decrease from 57.0 to 51.2. This demonstrates that CSME enhances multi-scale feature extraction while reducing model complexity. When only the EMSFFN module is introduced, the recall increases to 86.3% and mAP50 to 87.6%, indicating that the improved multi-scale feature fusion strategy effectively integrates features across different levels. However, the introduction of additional fusion structures leads to a slight increase in model complexity. When only the ASSA-AIFI module is applied, the recall and mAP50 improve to 86.2% and 87.4%, respectively. This module enhances key feature modeling through an adaptive sparse attention mechanism, suppressing redundant background information and enabling the model to better focus on defect regions.

Further analysis of different module combinations reveals strong complementarity among the proposed components. When CSME and EMSFFN are combined, the precision reaches 90.6% and mAP50 increases to 89.0%, demonstrating effective synergy between feature extraction and multi-scale fusion. When CSME is combined with ASSA-AIFI, the mAP50 reaches 88.5%, indicating that attention mechanisms further enhance feature representation. When EMSFFN and ASSA-AIFI are used together, the recall improves to 87.2%, suggesting reduced missed detections in complex scenarios.

Finally, when all three modules are integrated, the model achieves the best overall performance, with precision, recall, mAP50, and mAP50:95 reaching 92.1%, 87.6%, 89.4%, and 68.9%, respectively. At the same time, the model maintains a relatively low complexity, with only 15.9 MB parameters and 52.4 GFLOPs. In summary, all proposed modules contribute positively to wind turbine blade defect detection, and their collaborative integration significantly improves detection performance under complex backgrounds and multi-scale scenarios, validating the effectiveness of the proposed method.

## 5. Discussion

This study focuses on wind turbine blade surface defect detection in intelligent wind power maintenance scenarios. Due to large variations in defect scales and severe background interference, conventional detection methods often suffer from missed detections and false positives, which limit their practical applicability. To address these challenges, the proposed CEA-DETR enhances edge detail extraction, local texture representation, and multi-scale feature fusion. Experimental results demonstrate that CEA-DETR achieves superior performance compared to existing detection models, with Precision and Recall reaching 92.1% and 87.6%, respectively, and mAP50 and mAP50:95 achieving 89.4% and 68.9%. Compared with the baseline RT-DETR-r18, the proposed method improves Precision by 4.8%, Recall by 4.2%, and mAP50 and mAP50:95 by 3.1% and 6.5%, respectively, while reducing parameter count and computational cost by 20.1% and 8.1%. These results indicate that CEA-DETR effectively balances detection accuracy and computational efficiency, making it promising for deployment in resource-constrained inspection scenarios.

### 5.1. Theoretical Interpretability of Background Noise Filtering in ASSA-AIFI 

In practical wind turbine blade inspection scenarios, captured images typically contain complex backgrounds such as sky, clouds, vegetation, and tower structures. These background elements may introduce redundant feature interactions during attention computation, thereby degrading detection robustness, especially when defect boundaries are weak or visually similar to surrounding textures. From a theoretical perspective, conventional Transformer-based self-attention mechanisms compute pairwise correlations across all spatial positions, which inherently leads to dense feature aggregation. This dense connectivity, although beneficial for global context modeling, also increases the risk of propagating irrelevant background information, thereby acting as a form of noise amplification in complex visual environments. To address this limitation, the proposed ASSA-AIFI module introduces a hybrid attention mechanism that integrates sparse and dense attention branches. Specifically, the sparse self-attention branch applies a squared ReLU activation function to the attention map, which effectively suppresses low-response interactions. This operation can be interpreted as an adaptive filtering process that removes weak correlations likely associated with background noise, thereby enforcing sparsity in feature interactions. Meanwhile, the dense self-attention branch preserves full global dependencies, ensuring that long-range contextual relationships between spatially separated defect regions are maintained. The adaptive fusion of these two branches enables the model to simultaneously retain global contextual awareness while selectively emphasizing highly relevant features. Therefore, the ASSA-AIFI module can be interpreted as a balance between information preservation and noise suppression: the dense branch guarantees completeness of feature interactions, while the sparse branch acts as a noise filter by pruning redundant connections. This complementary design improves the signal-to-noise ratio of feature representations, leading to more discriminative attention distributions in complex environments. In addition, the CSME backbone further enhances defect-related features through multi-scale edge enhancement and dual-domain feature selection. Since many blade defects exhibit subtle edge characteristics, the reinforcement of high-frequency edge information improves boundary localization and reduces the risk of missed detections. Together, these mechanisms provide a theoretically grounded explanation for the improved robustness of the proposed model under complex background conditions.

### 5.2. Limitations of the Study

Despite the significant improvements achieved by CEA-DETR in wind turbine blade surface defect detection, several limitations should be acknowledged. First, the dataset used in this study is relatively limited in scale and diversity. Although the proposed model demonstrates strong performance on the collected dataset, its generalization capability under significantly different environmental conditions remains to be further validated. Second, the images in this study were primarily collected under relatively stable inspection conditions. In real-world wind farm environments, UAV-based inspection may encounter challenging conditions such as rain, fog, strong illumination variations, and motion blur caused by wind disturbances. These factors may degrade image quality and adversely affect detection performance. Finally, although the proposed model reduces computational complexity compared to the baseline RT-DETR, deploying the model on resource-constrained edge devices mounted on UAVs may still present challenges, particularly in terms of power consumption and thermal management during long-duration real-time inspections.

## 6. Conclusions

In this study, an improved RT-DETR-based framework, termed CEA-DETR, is proposed for wind turbine blade surface defect detection. By integrating the CSME backbone, the EMSFFN module, and the ASSA-AIFI mechanism, the proposed method enhances multi-scale feature representation, strengthens edge detail extraction, and improves the robustness of feature interaction under complex background conditions. Extensive experimental results demonstrate that CEA-DETR achieves superior detection performance compared with several mainstream detection models. In particular, it significantly improves detection accuracy while reducing model parameters and computational complexity relative to the RT-DETR-r18 baseline. These results indicate that the proposed framework provides an effective and efficient solution for accurate defect detection under complex visual conditions.

From a practical perspective, the proposed CEA-DETR framework shows strong potential for integration into UAV-based intelligent inspection systems for wind farms. By enabling reliable and efficient detection of blade surface defects, the method can support automated maintenance workflows, reduce manual inspection costs, and contribute to improving the safety, reliability, and operational efficiency of wind power infrastructure. More broadly, this work provides a feasible technical pathway toward intelligent and scalable inspection systems in industrial environments.

Despite these promising results, several limitations should be acknowledged. First, the dataset used in this study is relatively limited in scale and diversity, which may affect the generalization capability of the model in unseen scenarios. Second, the model has not been extensively validated under extreme environmental conditions, such as severe weather or drastic illumination variations, which may introduce additional challenges in real-world deployment. Nevertheless, the consistent performance improvements observed across multiple evaluation metrics indicate the robustness and effectiveness of the proposed method within the current experimental setting.

Future work will focus on several key directions. First, larger and more diverse datasets will be constructed to improve model generalization and robustness. Second, lightweight optimization techniques, including structured pruning and knowledge distillation, will be investigated to further reduce computational overhead. In addition, deployment-oriented evaluation, such as end-to-end latency testing on edge devices, will be conducted to validate practical applicability. Finally, the proposed modules, particularly the CSME backbone, will be extended to other high-precision industrial defect detection tasks to further verify their generalization capability and practical value.

## Figures and Tables

**Table 1:** Results of comparative experiments on different backbone networks.

Model	P/%	R/%	Parameters/MB	mAP50/%
ResNet18	87.3	83.4	19.9	86.3
ResNet50	88.0	83.8	43.1	86.9
FasterNet	87.1	82.6	14.6	85.7
ManbaOut	86.4	82.2	15.9	83.4
SwinTransformer	87.9	84.0	36.5	86.4
EfficientViT	86.8	83.5	14.1	86.6
CSME	89.5	85.9	15.8	88.2

**Table 2:** Results of comparative experiments on different feature fusion networks.

Model	P/%	R/%	Parameters/MB	mAP50/%
CCFM	87.3	83.4	19.9	86.3
SilmNeck	86.4	83.2	19.4	86.1
BiFPN	87.1	83.5	20.6	86.2
GDNeck	87.6	85.8	22.3	86.6
MAFPN	87.8	86.0	22.9	87.1
EMSFFN	88.4	86.3	20.1	87.6

**Table 3:** Results of comparative experiments on different attention module.

Model	P/%	R/%	Parameters/MB	mAP50/%
HiLo	87.6	83.6	20.1	86.4
EAA	87.4	84.7	20.2	86.2
DHSA	86.3	83.2	20.2	85.8
Pola	87.8	85.4	20.3	86.7
ASSA	88.2	86.2	20.7	87.4

**Table 4:** Comparison of detection performance of different models.

Model	AP50/%						mAP50/%	mAP50:95/%	Params/MB	GFLOPs	FPS
	Crack	Burning	Peel	Deformity	Rusty	Dirt					
Faster R-CNN	76.3	77.4	79.8	82.9	81.7	79.2	79.6	59.0	41.4	143.5	32.3
YOLOv5m	80.3	83.6	82.3	88.6	86.9	86.2	84.7	60.2	21.3	49.2	103.5
YOLOv7	81.8	81.5	82.6	88.1	85.3	86.5	84.3	62.1	36.5	103.7	53.7
YOLOv8m	82.2	82.6	84.8	90.3	86.9	88.6	85.9	63.3	25.8	78.7	79.9
YOLO11m	81.3	82.1	84.0	89.1	86.4	88.2	85.2	62.8	20.1	67.6	90.6
YOLOv12m	80.5	82.8	83.5	89.4	85.1	87.8	84.9	62.6	20.2	67.1	91.3
RTDETR-r18	83.1	84.6	85.2	90.2	86.1	88.7	86.3	62.4	19.9	57.0	65.5
RTDETR-r50	84.2	85.1	86.6	90.4	87.3	89.1	87.1	65.7	42.1	136.3	37.6
Deformable-DETR	83.5	84.8	85.2	90.1	86.6	89.0	86.5	64.8	40.0	173.2	33.2
CEA-DETR	86.7	87.7	90.5	90.5	90.9	90.2	89.4	68.9	15.9	52.4	63.2

**Table 5:** Ablation experiments results of DSM.

SSM	FSM	P	R	mAP50/%	Params/M	GFLOPs
		88.7	84.3	87.1	14.9	50.3
√		88.9	84.7	87.3	15.0	50.8
√	√	89.5	85.9	88.2	15.1	51.2

**Table 6:** Ablation experiment results of CEA-DETR.

CSME	EMSFFN	ASSA-AIFI	P	R	mAP50/%	mAP50:95/%	Params/M	GFLOPs
			87.3	83.4	86.3	62.4	19.9	57.0
√			89.5	85.9	88.2	66.3	15.1	51.2
	√		88.4	86.3	87.6	65.7	20.1	57.6
		√	88.2	86.2	87.4	65.1	20.7	58.8
√	√		90.6	87.1	89.0	68.2	15.1	51.8
√		√	90.3	86.8	88.5	67.8	15.8	52.1
	√	√	89.7	87.2	88.3	66.9	21.1	58.8
√	√	√	92.1	87.6	89.4	68.9	15.9	52.4

## Data Availability

The original contributions presented in this study are included in the article. Further inquiries can be directed to the corresponding author.
